# Tetracyclic Bis-Piperidine Alkaloids: Structures, Bioinspired Synthesis, Synthesis, and Bioactivities

**DOI:** 10.3390/molecules30142907

**Published:** 2025-07-09

**Authors:** Stan Iridio Gómez, Esveidy Isabel Oceguera Nava, Abbas Dadawalla, Dennis Ashong, Guanglin Chen, Qiao-Hong Chen

**Affiliations:** Department of Chemistry & Biochemistry, California State University, Fresno, CA 93740, USA

**Keywords:** natural product, piperidine alkaloids, chemical structure, synthesis, bioinspired synthesis, anticancer activity

## Abstract

Tetracyclic bis-piperidine alkaloids (TcBPAs) are structurally complex natural products primarily isolated from marine sponges of the order Haplosclerida. Distinguished by their intricate architecture, TcBPAs feature two central piperidine units linked by dual macrocyclic rings. These unique structural motifs contribute significantly to their biological activities. For example, TcBPAs exhibit antiproliferative activities at low micromolar concentrations across various cancer cell lines, including leukemia, melanoma, breast, colon, fibrosarcoma, and glioblastoma. Despite this promising therapeutic profile, the structural intricacy of TcBPAs has posed considerable challenges to the development of efficient synthetic methodologies, thereby limiting comprehensive exploration and potential clinical advancement. This review highlights recent progress and persisting challenges in the synthesis, structural analysis, and biological evaluation of TcBPAs, underscoring their therapeutic potential in anticancer drug discovery.

## 1. Introduction

Natural products have served as vital medicinal resources dating back as early as 2600 BC; particularly valued for their therapeutic potential against numerous diseases, notably cancer [1]. Their structural uniqueness and diverse biological activities have historically driven the discovery and development of novel therapeutics, often bypassing conventional screening limitations [2]. Reflecting their persistent significance, natural products or their derivatives accounted for 441 out of 1394 small-molecule drugs approved by the FDA between 1981 and 2019 [3].

Among the diverse classes of natural products, alkaloids represent an especially important group of nitrogen-containing secondary metabolites known for their unique structural diversity and significant medicinal properties. Alkaloids have been utilized medicinally for more than 4,000 years [2], and comprise approximately 20% of all plant-derived secondary metabolites. They play essential ecological roles, protecting plants from predators and assisting in growth regulation [2,4]. The term “alkaloid”, coined in 1819 by German chemist Carl F.W. Meissner, originates from the Arabic word al-qali, associated with a soda-producing plant [5]. Alkaloids demonstrate a wide range of therapeutic activities such as anesthetic, cardioprotective, anticancer, antimalarial, and anti-inflammatory effects [6]. Although predominantly found in plants, alkaloids have also been isolated from fungi, marine sponges, and animals, with clinically relevant examples including morphine, quinine, nicotine, and strychnine. 

**Figure 1:**
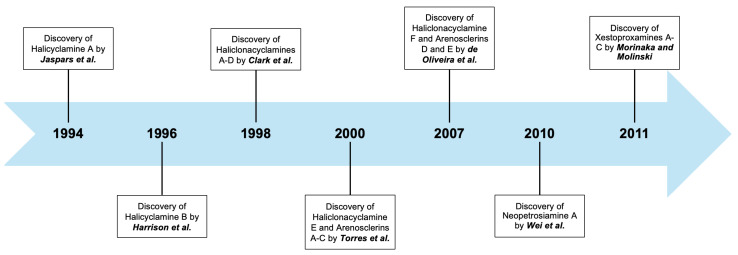
Timeline of the Discovery of TcBPAs [8,9,10,11,12,13,14].

Within alkaloids, the tetracyclic bis-piperidine alkaloids (TcBPAs) have emerged as a particularly intriguing subgroup, predominantly isolated from marine sponges within the family Haplosclerida [7]. A timeline highlighting key milestones in the discovery of TcBPAs is presented in Figure 1. First identified from marine sponge extracts in the 1990s [8], TcBPAs are characterized by their distinctive bis-piperidine units integrated into tetracyclic macrocyclic frameworks. The various subclasses of TcBPAs, including halicyclamines, haliclonacyclamines, and arenosclerins, differ primarily in ring size, saturation, and stereochemistry [9]. TcBPAs have gained particular interest due to their selective antiproliferative activities against leukemia and melanoma cell lines, demonstrating potent low micromolar effects and highlighting their potential as anticancer lead compounds. This review comprehensively explores recent advancements regarding TcBPAs, with a detailed focus on their chemical structures, biosynthetic hypotheses, synthetic methodologies, and biological activities, emphasizing their therapeutic relevance and future promise.

## 2. General Structural Features, Natural Occurrence, and Stereochemistry 

### 2.1. General Structural Features

**Figure 2:**
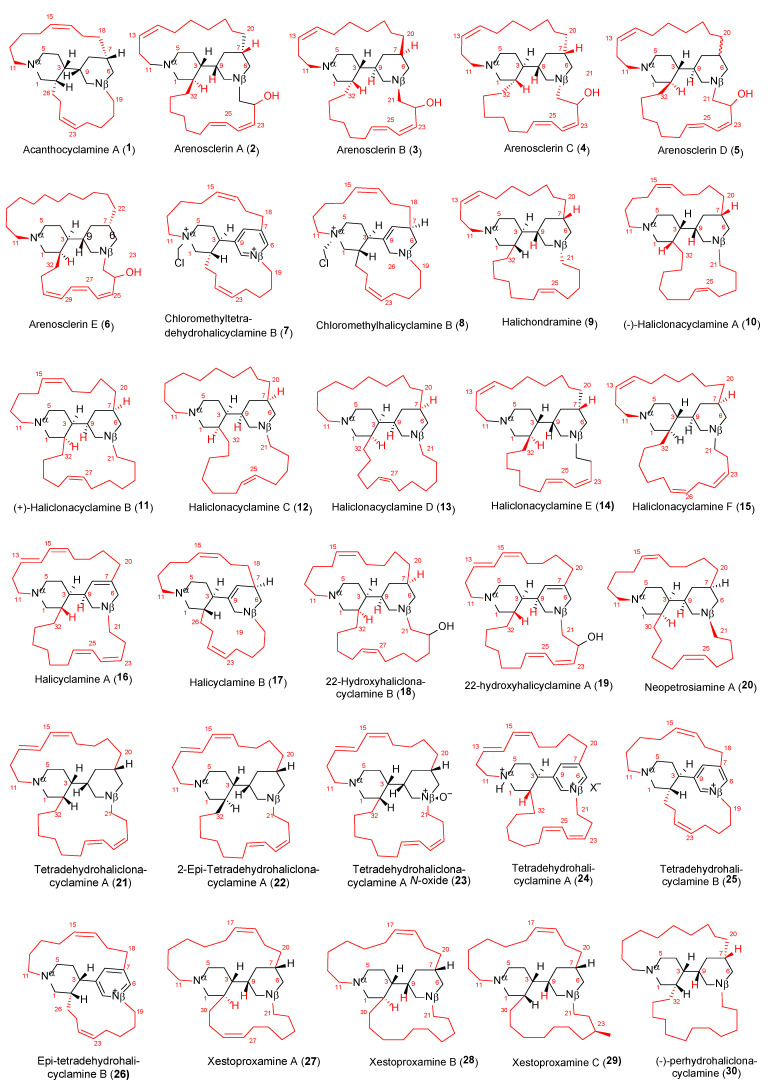
Chemical structures of 27 Naturally Occurring TcBPAs (**1–6**, **9–29**), Two Isolation Artifacts (**7** and **8**), and One Semi-Synthetic Derivative (**30**).

TcBPAs are defined by a central 3,9-linked bis-piperidine core, flanked by two aliphatic macrocyclic chains. This core features two substituted piperidine rings connected via two hydrocarbon linkers that vary in length, degree of unsaturation, and functionalization. The resulting structure forms a fused heterocyclic–carbocyclic tetracyclic framework containing two nitrogen atoms. To date, 27 naturally occurring TcBPAs (**1–6**, **9–29**, Figure 2) have been isolated from marine sponges, along with two chloromethylated derivatives (**7** and **8**) identified as isolation artifacts resulting from reaction with dichloromethane. These compounds share a conserved tetracyclic bis-(3-alkylpiperidine) skeleton but differ in spacer chain composition and stereochemistry in the bis-piperidine core. The aliphatic bridges typically range from 8 to 12 carbon atoms and may contain zero to three double bonds, whose number and location contribute to the structural and functional diversity within the class. For example, haliclonacyclamines A–F (**10–15**) generally possess C10 and C12 linkers, while halicyclamine B (**17**) features two shorter C8 chains. Neopetrosiamine A (**20**) contains one C10 and one C12 chain, each with internal *Z*-configured double bonds at Δ^15,16^ and Δ^25,26^. Similarly, halicyclamine A (**16**) includes conjugated dienes in both linkers, and arenosclerin E (**6**) is distinguished by an unusual (25*Z*,27*Z*,29*Z*)-triene motif in its C10 bridge.

Several TcBPAs also incorporate polar substituents within their side chains. Arenosclerins A–E (**2–6**) and 22-hydroxyhaliclonacyclamine B (**18**) contain a hydroxyl group along the alkyl spacer, while xestoproxamine C (**29**) uniquely features a methyl substituent in the bridge. These structural variations expand the functional diversity and potential bioactivity of the TcBPA scaffold. Structurally, the haliclonacyclamines (**10–15**) and halicyclamines (**16** and **17**) can be differentiated by their bis-piperidine stereochemistry and by the number and positioning of double bonds within the piperidine rings. However, the reported stereochemistry of the bis-piperidine core often varies across compounds (see Section 2.3), bringing to light the challenges in assigning absolute configuration in this compound class. The arenosclerins (A–D) (**2–5**), first described by Torres et al. in 2000 from *Arenosclera brasiliensis*, are considered derivatives of haliclonacyclamine E (**14**), differing in the stereochemistry of the bis-piperidine unit [10]. These compounds consistently carry a 22-hydroxy substituent, with arenosclerin E (**6**) further distinguished by the additional double bond in the southern alkyl bridge [10,15].

Importantly, chloromethylhalicyclamine B (**8**) was later identified as a solvent-derived artifact formed from halicyclamine B (**17**) during prolonged storage in dichloromethane. LC-MS analysis demonstrated approximately 40% conversion after 14 days, while the absence of compound **8** in butanol extracts confirmed its non-natural origin [16]. Finally, perhydrohaliclonacyclamine (**30**) represents the fully saturated analog of the haliclonacyclamine family (**10–15**). Although not isolated directly from nature, it was obtained via catalytic hydrogenation of haliclonacyclamine E (**14**), originally isolated from *A. brasiliensis* [10]. This semi-synthetic derivative was instrumental in elucidating absolute stereochemistry across the series using circular dichroism (CD) analysis, as reported by Morinaka et al. [13].

### 2.2. Natural Occurrence and Geographic Distribution of TcBPAs

TcBPAs are a distinctive family of marine natural products primarily isolated from sponges within the order Haplosclerida, including genera such as *Haliclona*, *Xestospongia*, *Petrosia*, and *Neopetrosia*. The occurrence of 3-alkylpiperidine compounds is often considered a chemotaxonomic marker for the Haplosclerida order of sponges [17]. Table 1 summarizes 27 naturally occurring TcBPAs (**1–6**, **9–29**) isolated from marine sponges, along with two chloromethylated derivatives (**7** and **8**) identified as isolation artifacts, including their corresponding source organisms and collection sites. So far, *Acanthostrongylophora ingens*, *Haliclona* spp. and *Arenosclera brasiliensis* are the most prolific taxa. *A. ingens* is the reported source of seven alkaloids—including acanthocylamine A (**1**), halicyclamine B (**17**), and several chlorinated or dehydrogenated derivatives—all obtained from sponge specimens collected off the coast of South Sulawesi, Indonesia. These compounds share bispiperidine or halicyclamine-type frameworks, indicating a biosynthetically active genus in this region. *Haliclona species* collected from Heron Island, Australia, have yielded haliclonacyclamines A–D (**10–13**), while *Arenosclera brasiliensis* and *Pachychalina alcaloidifera*, both marine sponges collected from the southeastern coast of Brazil, have produced arenosclerins (**2–6**) and additional haliclonacyclamine analogs. These findings reflect the diversity of TcBPAs associated with specific sponge taxa and local habitats. In addition, *Neopetrosia proxima*—collected from Mona Island, Puerto Rico, and the Bahamas—has yielded multiple TcBPAs, including neopetrosiamine A (**20**) and xestoproxamines A–C (**27**–**29**). These occurrences further illustrate the taxonomic range of TcBPA-producing sponges and the variety of marine environments from which they have been isolated.

While certain TcBPA types, such as the halicyclamines (**16** and **17**), have been isolated from multiple genera and locations, others—including the arenosclerins (**2–6**)—appear more taxonomically and geographically limited based on current reports. These observations point toward both shared and lineage-specific biosynthetic capacities among marine sponges. 

### 2.3. Stereochemistry

As noted earlier, twenty-nine TcBPAs have been isolated from marine sponges. These compounds exhibit significant stereochemical diversity, particularly in the relative and absolute configurations of the associated methine carbons in the piperidine ring systems. Their reported optical rotations and configurations are summarized in Table 2. It is worth noting that several TcBPAs have been shown to be enantiomerically pure based on chiral HPLC analyses.

The absolute configurations of eleven TcBPAs have been conclusively established using a combination of X-ray crystallography, Electronic Circular Dichroism (ECD), and integrated spectroscopic techniques. In many cases, X-ray crystallography, frequently utilizing anomalous dispersion (resonant scattering), facilitates direct stereochemical determination. This technique involves slight deviations from Friedel’s Law, essential for establishing absolute structures in non-centrosymmetric crystals, crucial for determining absolute configurations in organic molecules. Although historically challenging for light-atom compounds (e.g., C, N, and O), recent advancements, including Bayesian-based Bijvoet analysis (Hooft method), meticulous low-temperature data collection, and optimized X-ray wavelengths (e.g., Cu-Kα radiation), have significantly enhanced accuracy [25]. This approach was used to assign the configurations of haliclonacyclamines A and B (**10** and **11**), acanthocyclamine A (**1**) (all 2R,3R,7R,9R), tetradehydrohaliclonacyclamine A (**24**), and (–)-perhaliclonacyclamine (**30**) (both 2S,3S,7S,9S).

For other TcBPAs, including halicyclamine B (**17**) and the xestoproxamines (**27–29**), absolute configurations were determined via ECD and chiroptical analyses. Xestoproxamines A and B (**27** and **28**) were assigned a 2R,3S,7S,9S configuration, while Xestoproxamine C (**29**) bears an additional stereocenter (2S,3S,7S,9S,23S). Arenosclerin A (**2**) and haliclonacyclamine E (**14**) were also assigned the 2R,3S,7S,9S configuration using exciton-coupled CD (ECCD) analysis of their bis-p-bromophenacyl derivatives, referenced against (–)-perhaliclonacyclamine (**30**). The latter compound, a fully saturated derivative of the tetradehydrohaliclonacyclamines, played a pivotal role in validating the stereochemistry of related alkaloids through chiroptical comparison [13,24].

In addition to absolute assignments, the relative configurations of other TcBPAs were elucidated by analyzing X-ray crystallographic analysis, ^1^H NMR coupling constants, and dipolar interactions observed in NOESY and ROESY spectra. These combined analytical approaches have significantly advanced the stereochemical understanding of this unique class of marine alkaloids.

The stereochemistry at the bridgehead nitrogen atoms (e.g., N_α_ and N_β_) in TcBPAs is inconsistently depicted in the literature, and their absolute configuration has often not been rigorously assigned. While tertiary amines are formally chiral centers, their stereochemical stability is highly dependent on structural constraints. In rigid polycyclic frameworks such as those found in TcBPAs, ring strain may hinder pyramidal inversion, thereby rendering the nitrogen centers configurationally stable. However, very little was discussed in the original references. Previous studies have also reported challenges in resolving stereochemistry due to overlapping ^1^H NMR signals and conformational flexibility of the piperidine rings [7,8,18]. As such, stereochemical drawings across different reports vary.

## 3. Biosynthetic Hypotheses and Bioinspired Syntheses

### 3.1. The Bioinspired Synthesis of TcBPAs Based on the Baldwin–Whitehead Hypothesis

The bioinspired synthesis of TcBPAs has attracted sustained interest due to their intricate molecular architectures and potent bioactivities. A unifying framework for understanding their biosynthetic origins is the influential Baldwin–Whitehead postulate [26], which proposes that these natural products are constructed from simple and readily available building blocks: ammonia, acrolein or its equivalent, and linear long-chain dialdehydes. These precursors condense to form a C_2_-symmetric achiral macrocyclic bis(dihydropyridine) intermediate, which undergoes an intramolecular endo-selective Diels–Alder cycloaddition. The resulting pentacyclic scaffold, often described as a keramaphidin-type intermediate (**35**), can then be elaborated into various structural families of alkaloids through skeletal rearrangements, bond cleavages, and reductions. A critical transformation in this model is the retro-Mannich-type fragmentation of a specific C2-C6 bond, leading to the formation of a tetracyclic core of TcBPAs [27]. This biosynthetic logic underpins the proposed pathways for halicyclamine A (**16**), haliclonacyclamine A (**10**), and acanthocyclamine A (**1**), each representing a variant on this central theme.

**Scheme 1:**
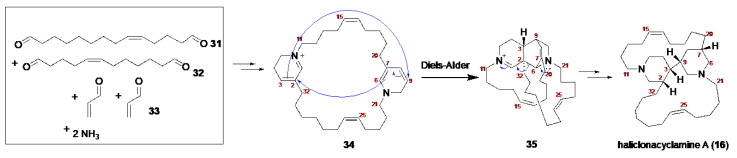
Proposed bioinspired synthetic pathway for halicyclamine A (**16**) [8].

The bioinspired synthetic pathway proposed for halicyclamine A (**16**) (Scheme 1) begins with the reductive coupling of two long-chain dialdehydes (**31** and **32**) with two equivalents each of acrolein (**33**) and ammonia. This sequence was proposed by Jaspars on the grounds of the Baldwin–Whitehead hypothesis [8]. This coupling generates a tricyclic precursor (**34**), which undergoes a Diels–Alder cyclization to yield a xestocyclamine/ingenamine-type structure (**35**) (Scheme 1) with a *cis* relationship between H-3 and H-9. A retro-Mannich-type fragmentation at the C2-C6 bond initiates a rearrangement that forms a C1-C2 double bond, which is stabilized by resonance and subsequently reduced to halicyclamine A. This pathway integrates early-stage aldehyde-ammonia condensation, endo-selective Diels–Alder cycloaddition, and late-state ring remodeling [26,28].

**Scheme 2:**
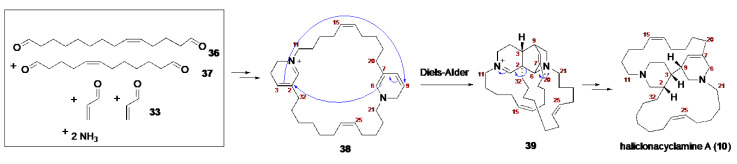
Proposed bioinspired synthetic pathway for haliclonacyclamine A (**10**) [20].

Similarly, the bioinspired synthetic pathway for haliclonacyclamine A (**10**), outlined by Charan [20] and shown in Scheme 2, follows the Baldwin–Whitehead paradigm. Two equivalents of acrolein (**33**) molecules condense with two long-chain (12 and 14 carbon atoms, respectively) monounsaturated dialdehydes (**36** and **37**) and two equivalents of ammonia to form a partially reduced bis(3-alkylpyridine) macrocycle (**38**). Diels–Alder cyclization sets the *cis*-H3/H9 stereochemistry in **39**, and an intramolecular rearrangement of **39** via a retro-Mannich-type fragmentation cleaves the C2-C6 bond, generating the haliclonacyclamine core. The final steps involve the reduction of the C6-*N* and C1-C2 double bonds formed during the rearrangement and hydrogenation of the C7-C8 double bond formed during cyclization.

**Scheme 3:**
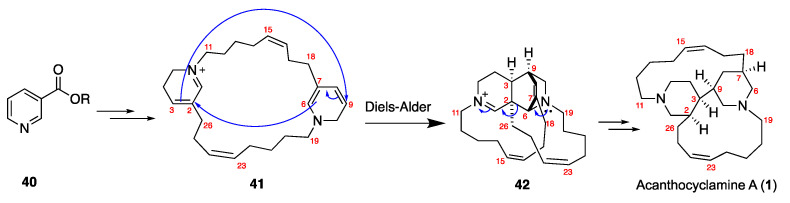
Proposed bioinspired synthetic pathway for acanthocyclamine A (**1**) [17].

In the case of acanthocyclamine A (**1**), the pathway proposed by Dewi et al. and illustrated in Scheme 3 [17] builds on earlier insights by Cutignano et al. [29]. Here the core precursor is a nicotinic acid derivative (**40**), which incorporates successive two-carbon units via the polyketide pathway. Following decarboxylation transformations, the growing chain forms a macrocyclic intermediate **41**, which undergoes an endo-Diels–Alder cyclization, generating a pentacyclic intermediate **42** in which H-3 and H-9 adopt a *cis* configuration. The final conversion to acanthocyclamine A (**1**) involves a ring-opening process that induces C2-C6 bond cleavage through a retro-Mannich-type fragmentation, the reduction of the resulting iminium cation, and the hydrogenation of the C1-C2 and C7-C8 double bonds. 

### 3.2. Alternative Bioinspired Synthetic Model: The Marazano Modification

**Scheme 4:**
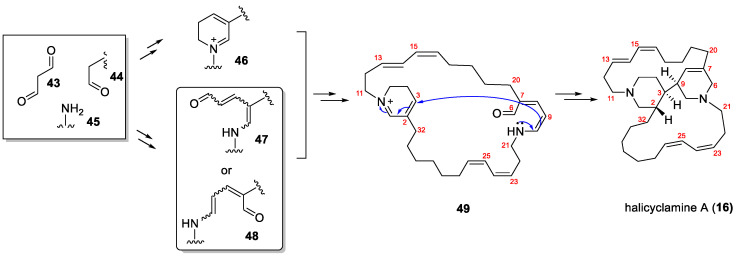
Bioinspired synthetic pathway of halicyclamine A (**16**) based on Marazano’s Proposal.

While the Baldwin–Whitehead postulate remains a foundational framework for understanding the bioinspired synthesis of TcBPAs, an alternative and mechanistically distinct model, developed by the Marazano research group, offers a compelling bioinspired synthetic route grounded in stepwise condensation chemistry and supported by experimental work [30,31,32,33]. This model (Scheme 4) proposes that aminopentadienal derivatives (**47** and **48**) play a central role as key bioinspired synthetic intermediates. These are envisioned to arise from the condensation of malondialdehyde (**43**), an aldehyde (**44**), and a primary amine (**45**), resulting in highly conjugated, nucleophilic intermediates. Unlike the Baldwin–Whitehead approach, which emphasizes Diels–Alder cyclization, this pathway relies on stepwise transannular 1,4-conjugate additions between the aminopentadienals (**47** and **48**) and cyclic iminium cations (**46**) derived from 5,6-dihydropyridinium salts. This reaction forms a new C-C bond and typically results in the generation of a pyridinium core, a structural feature present in several marine alkaloid families [27]. 

**Scheme 5:**
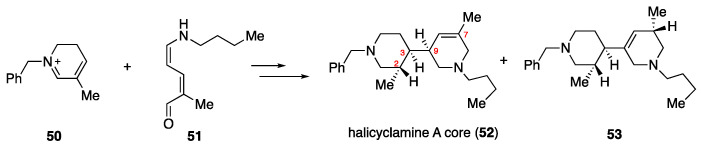
Biomimetic synthesis of the halicyclamine A core based on Marazano’s hypothesis.

In a key study, the Marazano research group [31] applied this model to the proposed bioinspired synthesis of halicyclamine A (**16**) (Scheme 4). They postulated that the macrocyclic intermediate **49,** containing both dihydropyridinium and aminopentadienal units, could form a polycyclic intermediate, representing an advanced bioinspired synthetic precursor to halicyclamine B and, by extension, halicyclamine A. To test this, they developed a model reaction (Scheme 5) where an aminopentadienal derivative (**51**) condenses with a partially reduced pyridinium salt (**50**) to yield an aminal. Subsequent acid treatment (methanesulfonic acid) and reduction (NaBH_4_) furnished adducts **52** and **53**, with compound **52** resembling the halicyclamine scaffold. They further proposed that partially reduced pyridinium salts like intermediate **50** may serve as bioinspired synthetic precursors to haliclonacyclamines A–C (**10**–**12**).

**Scheme 6:**
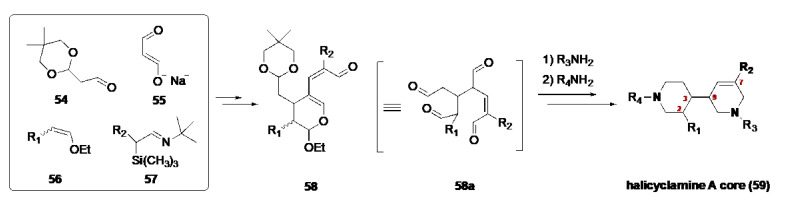
Biomimetic synthesis of the bicyclic core of halicyclamine A via a tetraaldehyde-based strategy.

Further support for this pathway comes from the work of the same research group [33], who pursued a biomimetic synthetic strategy aimed at accessing the bicyclic nucleus (**59**) of halicyclamine A (**16**, Scheme 6). Their synthetic design directly mimics their proposed biogenesis. It begins with the condensation of malondialdehyde derivatives (**54**, **55**), an enol ether **56**, and the anion of the silylimino derivative **57**, forming an intermediate **58** that serves as an equivalent to the key tetraaldehyde (**58a**), designed to mimic the natural precursor. Through a series of transformations, pyridinium salt formation, reduction to bicyclic amines, cyclization, and final reductive amination, the team synthesized bicyclic derivatives bearing *cis*- or *trans*-fused bispiperidine ring systems closely analogous to those in halicyclamine A (**16**) and other TcBPAs. This synthetic achievement reinforces the idea that malondialdehyde-derived tetraaldehydes could serve as a universal bioinspired synthetic precursor for structurally diverse marine alkaloids, including halicyclamines.

Taken together, the work of the Marazano research group presents a comprehensive and experimentally grounded bioinspired synthetic model that complements the Baldwin–Whitehead postulate. By shifting the emphasis from symmetry-controlled cycloadditions to nucleophile–electrophile couplings, transannular reactivity, and stepwise macrocyclization, this alternative model greatly expands the mechanistic possibilities for the bioinspired synthesis of complex marine alkaloids. 

### 3.3. Stereochemical and Experimental Insights into TcBPA Bioinspired Synthesis

The observed stereochemical diversity among TcBPAs provides key insights into their bioinspired synthetic origins. Several natural products such as acanthocyclamine A (**1**) exhibit high enantiomeric purity, with a defined configuration of (2R,3R,7R,9R) [17], while others like tetradehydrohaliclonacyclamine A (**21**) adopt the opposite enantiomeric series (2S,3S,7S,9S) [24]. Such stereochemical precision strongly suggests the involvement of an enantioselective enzymatic control, possibly by a Diels–Alderase or other stereodirecting enzymes [24]. However, the co-isolation of related 3-alkylpiperidine alkaloids as racemates or non-equimolar enantiomeric mixtures indicates that not all bioinspired synthetic steps are uniformly enzyme-catalyzed, or that enzymatic selectivity may vary across species or intermediates [27,34]. For example, the opposite absolute configurations observed in tetradehydrohaliclonacyclamine A (**21**) and the haliclonacyclamine series (**10–15**), despite their shared structural frameworks, support the idea that the bioinspired synthetic pathways can give rise to both enantiomeric series, potentially through non-enzymatic or steps prone to epimerization [24].

The co-occurrence of structurally diverse alkaloids, including madangamines, haliclonacyclamines, arenosclerins, ingenamines, and cyclostellettamines, in a single sponge species provides compelling support for a common biogenetic origin [9,24]. Such diversity implies that nature may rely on a shared biosynthetic framework, where slight modifications to macrocyclization, substitution patterns, or oxidation states give rise to multiple alkaloid families from similar precursors. Efforts to mimic these complex biosynthetic transformations in vitro have historically faced significant challenges. For example, biomimetic attempts to replicate key cyclization events often yield the desired products in low yields (e.g., 0.2–0.3% for keramaphidin B), hampered by competing side reactions such as disproportionation [27,30,32,35,36].

## 4. Chemical Synthesis

Various synthetic strategies and biomimetic approaches have been attempted to access the TcBPAs. These approaches are often informed by proposed biosynthetic pathways.

### 4.1. Total Synthesis of Halichonacyclamine C (***12***) and Tetrahydrohaliclonacyclamine A (***89***)

**Scheme 7:**
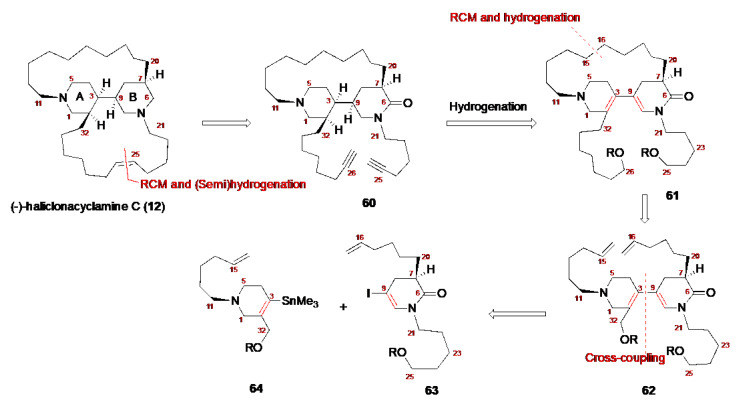
Sulikowski’s Retrosynthetic Analysis of Haliclonacyclamine C (**12**).

A pioneering total synthesis of TcBPAs was reported by Smith and Sulikowski at Vanderbilt University in 2010 for racemic haliclonacyclamine C (**12**) and its derivative tetrahydrohaliclonacyclamine A (**89**) [37,38]. The convergent retrosynthetic analysis, as shown in Scheme 7, relied on assembling two key fragments: an iodoenamine (**63**) and an alkylated vinyl stannane (**64**). These fragments were coupled via a Stille reaction to afford the bicyclic tetraene precursor (**62**). The fragment C26-C32 was incorporated by a Tsuji-Trost reaction. The C15-C16 bond was constructed via an olefin metathesis using Grubb’s first-generation catalyst followed by catalytic hydrogenation to yield tricyclic intermediate **61**. The terminal diyne functionality was constructed by oxidizing the primary alcohol groups in **61** with Dess–Martin periodinane followed by reacting with Bestmann–Ohira reagent. Subsequent reduction with Red-Al yielded diamine diynes, which underwent methylation and reaction with sodium thiophenoxide to produce methylated diamine intermediate **60**. This precursor was then subjected to a molybdenum-catalyzed alkyne metathesis followed by a Lindlar reduction to yield the target alkaloid haliclonacyclamine C (**12**). 

**Scheme 8:**
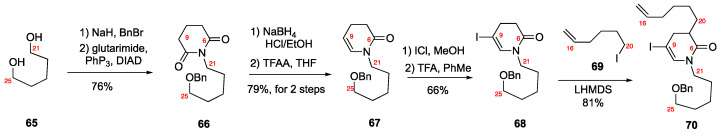
Sulikowski’s synthesis of iodoenamine precursor **70**.

The synthesis of the iodoenamine precursor (**70**), as illustrated in Scheme 8, begins with the benzylation of diol **65** to protect one of its hydroxyl groups, yielding 5-benzyloxypentanol. A subsequent Mitsunobu reaction with glutarimide installs the imide moiety, affording glutarimide **66**, which is then reduced with sodium borohydride to give an alcohol. Elimination of the hydroxyl group mediated by trifluoroacetic anhydride (TFAA) furnishes the corresponding enamine **67**. The treatment of enamine **67** with iodine monochloride in methanol induces a regioselective iodomethoxylation, resulting in a methoxyiodo amide intermediate. Subsequent brief exposure to catalytic trifluoroacetic acid (TFA) in refluxing toluene promotes the elimination of methanol, furnishing the iodoenamide **68** via the re-establishment of the enamine double bond. In the final step, compound **68** is deprotonated with LiHMDS in THF to generate a nucleophilic species, which undergoes electrophilic substitution with 6-iodo-1-hexene (**69**), delivering the targeted iodoenamine precursor **70**. This multistep route results in an overall yield of 31% [37,38].

**Scheme 9:**
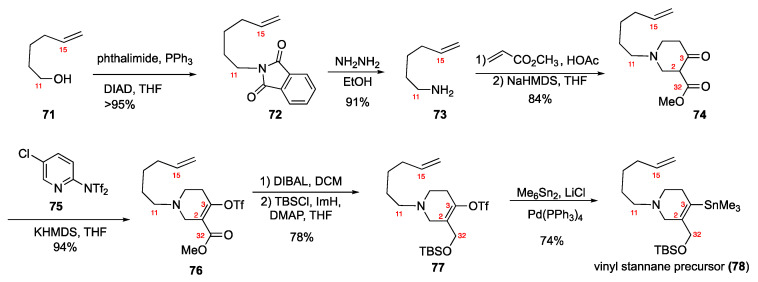
Sulikowski’s synthesis of vinyl stannane precursor **78**.

As illustrated in Scheme 9, the synthesis of the vinyl stannane precursor **78** begins with a Mitsunobu reaction between 5-hexen-1-ol (**71**) and phthalimide, affording compound **72**. The treatment of **72** with hydrazine in ethanol cleaves the phthalimide group, yielding primary amine **73**. This intermediate undergoes two sequential Michael additions, followed by intramolecular cyclization promoted by NaHMDS to furnish β-keto ester **74**. Subsequent triflation with **75** affords compound **76**, which is reduced with DIBAL-H followed by hydroxyl protection to produce compound **77**. A final palladium-catalyzed stannylation using hexamethyldistannane provides the desired vinyl stannane precursor **78**. The overall yield for this eight-step sequence is 29% [37,38].

**Scheme 10:**
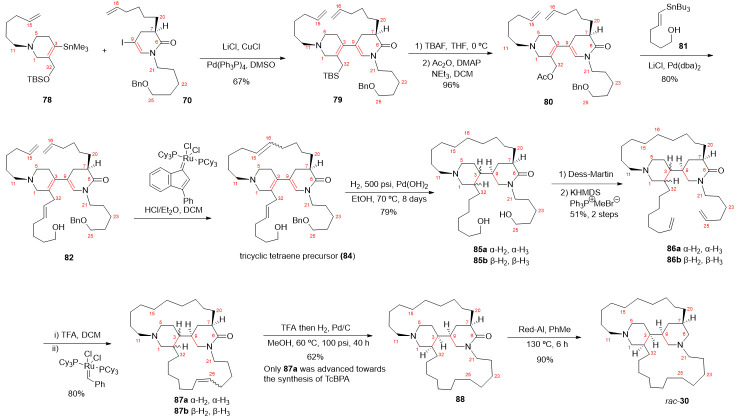
Completion of tetrahydrohaliclonacyclamine A (*rac*-**30**).

As shown in Scheme 10, the two key precursors, iodoenamine **70** and vinyl stannane **78**, underwent a Stille cross-coupling to afford intermediate **79**, which upon the removal of the TBS protecting group followed by acylation yielded **80**. A subsequent Tsuji-Trost reaction of **80** with **81** produced the ring-closing metathesis substrate **82**, a diene that was subjected to ring-closing alkene metathesis mediated by catalyst **83** to form the tricyclic tetraene precursor **84**. This compound was converted to its TFA salt and then exposed to hydrogenation using Pearlman’s catalyst under 500 psi of hydrogen at 70 °C for 8 days, resulting in an inseparable 1.3:1 mixture of two diastereomers, **85a** and **85b** [37,38]. The non-stereoselective hydrogenation at the C2-C3 double bond is likely due to stereochemical flexibility of the adjacent tertiary amine. The primary alcohols in these diastereomeric mixtures were oxidized to aldehydes using Dess–Martin periodinane, followed by Wittig olefination with methylenetriphenylphosphorane (10 equiv) to furnish a mixture of terminal dienes, **86a** and **86b**. These dienes, in their TFA salts, were subjected to Grubbs’ first-generation catalyst-mediated olefin metathesis, giving rise to two separable tetracyclic isomers, **87a** and **87b**. The isomers were isolated individually by flash chromatography, and only **87a** was advanced toward the synthesis of TcBPA. Hydrogenation of TFA salt **87a** at 100 psi and 60 °C for 40 h yielded the tetracyclic amide **88**, whose X-ray analysis confirmed the *cis*-syn-*cis* stereochemistry of the TcBPA core. Amide **88** was subsequently treated with Red-Al to furnish tetrahydrohaliclonacyclamine A (*rac*-**30**), the fully saturated analog of the natural product [37,38].

**Scheme 11:**
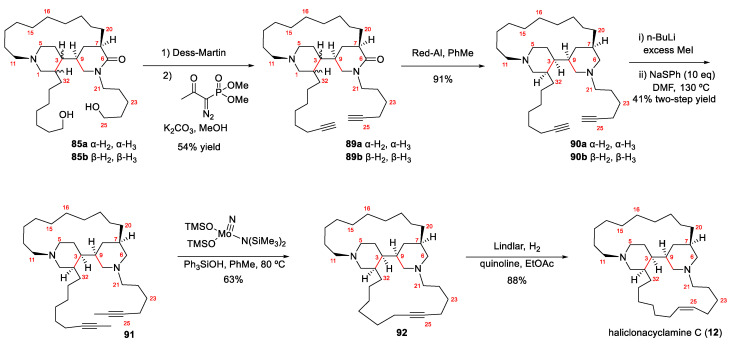
Completion of haliclonacyclamine C (**12**).

As outlined in Scheme 11, the synthesis of haliclonacyclamine C (**12**) began with the oxidation of isomers **85a** and **85b** using Dess–Martin periodinane to furnish the corresponding aldehydes, which were then treated with an excess of the Bestmann–Ohira reagent to yield the terminal diynes **89a** and **89b**. This mixture was subsequently reduced with Red-Al, affording a pair of diamine terminal diynes, **90a** and **90b**; only **90a** was carried forward. Treatment of diyne **90a** with n-butyllithium, followed by excess methyl iodide, led to the formation of the quaternary ammonium salt. This intermediate was then reacted with an excess of sodium thiophenoxide (10 equiv) in DMF at 130 °C, yielding the methylated diamine **91**. The resulting diamine was introduced into a molybdenum-based catalyst system, and the reaction was conducted at 80 °C for 2 h, achieving the first reported alkyne metathesis of this type to form the tetracyclic alkyne **92**. Finally, reduction of **92** with the Lindlar catalyst provided the fully saturated natural product haliclonacyclamine C (**12**).

### 4.2. Synthesis of Epi-Tetradehydrohalicyclamine B (***26***)

**Scheme 12:**
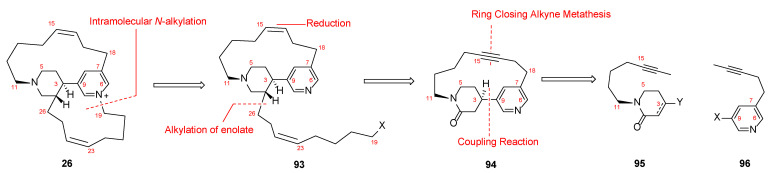
Dalling’s Retrosynthetic Analysis of epi-tetradehydrohalicyclamine B (**26**).

The first total synthesis of *epi*-tetradehydrohalicyclamine B (**26**), a rare tetracyclic marine pyridinium alkaloid, was reported by Dalling and Fürstner in 2022 [27]. Their innovative approach revitalized synthetic interest in the TcBPA family, demonstrating the effectiveness of modern catalytic strategies for challenging macrocyclic pyridinium frameworks. The retrosynthetic analysis (Scheme 12) utilized a convergent strategy involving four key transformations: (i) *N*-C19 bond formation via intramolecular *N*-alkylation of **93** (ii) C2-C26 bond formation in **94** via alkylation of an enolate, (iii) C3-C9 bond formation in **94** via Ni/Ir photoredox-catalyzed C-C bond construction of **95** and **96**, and (iv) macrocyclization through ring-closing alkyne metathesis (RCAM) to form a C15-C16 triple bond in **94**. Specifically, the synthesis began by establishing the bicyclic framework through a Ni/Ir dual photoredox catalysis, forming the critical C3-C9 bond under mild conditions. To build the tricyclic scaffold (**94**), a molybdenum-based alkylidyne catalyst was employed for RCAM, which successfully tolerated the presence of the pyridine nitrogen despite the inherent Lewis acidity of the Mo(VI) center. The C2-C26 bond in **93** was then introduced in the synthesis via alkylation of a suitably stabilized enolate. A challenging tertiary amide reduction was then achieved with Vaska’s complex and TMDS, yielding an enamine intermediate without affecting neighboring unsaturation or the sensitive heteroaromatic core. The final macrocyclization involved an intramolecular *N*-alkylation, forming the *N*-C19 bond and closing the 13-membered pyridinium ring. This was accomplished through deprotection of the TBDPS, iodination of the liberated primary alcohol, and thermal cyclization in refluxing acetonitrile. The transformation proceeded in 49% yield, with excellent site- and chemoselectivity, favoring the 13-membered product over a more strained and less favorable 11-membered ring.

**Scheme 13:**
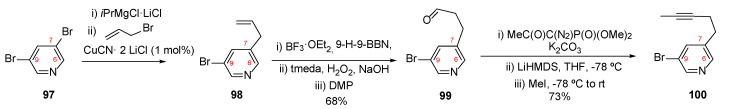
Synthesis of **100**.

Fragment **100** (equivalent to **96**) was synthesized from commercially available 3,5-dibromopyridine (**97**, Scheme 13) via Grignard reagent formation and copper-catalyzed alkylation to afford alkylated bromopyridine **98**. Subsequent hydroboration-oxidation and Dess–Martin oxidation provided aldehyde **99**, which was further converted into methylated alkyne precursor **100** via the Bestmann–Ohira homologation and methylation sequence.

**Scheme 14:**
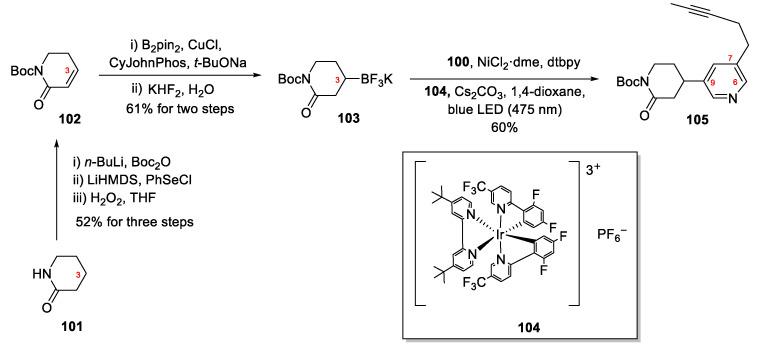
Synthesis of **105**.

Bicyclic precursor **105** (Scheme 14) originated from 2-piperidone (**101**). Boc-protection, α-selenation, oxidative elimination, and copper-catalyzed borylation provided trifluoroborate salt **103**, which underwent a crucial Ni/Ir dual-catalyzed cross-coupling with alkyne **100** to form intermediate **105**.

**Scheme 15:**
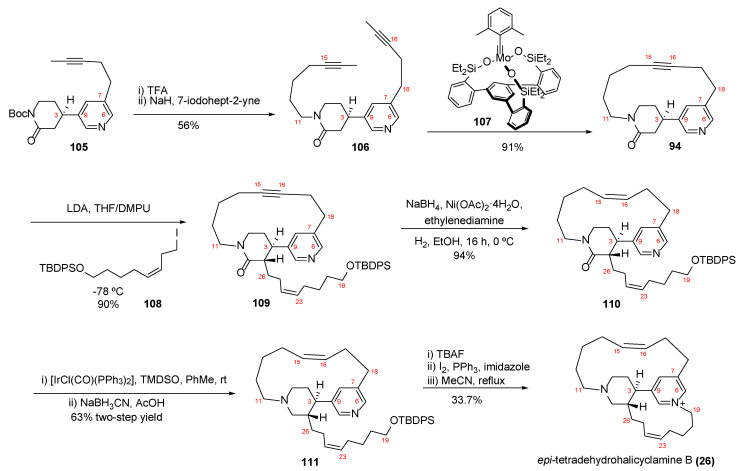
Synthesis of *epi*-tetradehydrohalicyclamine B (**26**).

The concluding synthesis stages (Scheme 15) involved deprotection of **105**, *N*-alkylation to produce intermediate **106**, and macrocyclization via RCAM using molybdenum catalyst **107** to obtain intermediate **94**. Subsequent transformations, including α-alkylation, selective *cis*-alkene hydrogenation, and lactam reduction, culminated in the final intramolecular *N*-alkylation step. This efficiently completed the total synthesis of *epi*-tetradehydrohalicyclamine B (**26**) in a cumulative 34% yield over the final three steps.

### 4.3. Dimerization of 1,6-Dihydropyridines Towards Halicyclamine Mimics

**Scheme 16:**
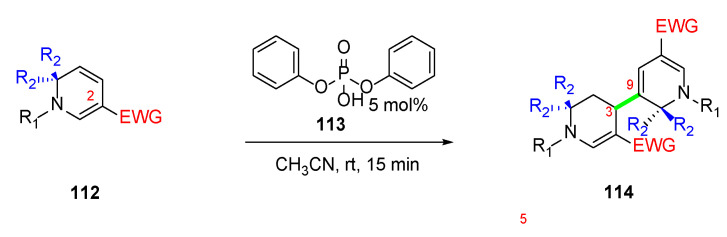
General Scheme of Substituted 1,6-DHP dimerization.

The successful synthesis of halicyclamine-type scaffolds via biomimetic transannular cyclization represents a breakthrough, spearheaded by Wayama and Oguri in 2022 and 2023 [35,36]. Earlier biomimetic strategies, inspired by the Baldwin–Whitehead hypothesis, envisioned transannular Diels–Alder cycloadditions of bis-1,6-DHP intermediates. However, these approaches were largely unsuccessful, producing only trace amounts (0.2–0.3%) of the desired cycloadducts. The main obstacle was disproportionation, driven by hydride shifts within the DHP framework, which diverted reactivity away from productive cyclization. Wayama and Oguri overcame these challenges by strategically redesigning the DHP structure and carefully tuning reaction conditions (Scheme 16). Two critical modifications were implemented: (1) geminal disubstitution at the C5 position, which eliminated hydride donors and suppressed disproportionation; and (2) incorporation of an electron-withdrawing group at C2, which directed regioselective C3 protonation and promoted C3-C9 bond formation in TcBPAs over undesired iminium reactivity at C1. These innovations enabled highly regioselective, metal-free dimerizations of 1,6-DHPs under mild Brønsted acid catalysis (Scheme 16). Specifically, diphenyl phosphate (**113**) catalyzed the dimerization in acetonitrile at room temperature, completing within 15 min. The optimized structural design and reaction setup led to the first successful dimerization of 1,6-DHPs (**112**) to form halicyclamine-type polycyclic frameworks (**114**), offering a generalizable strategy for the synthesis of TcBPA analogs.

### 4.4. Synthesis of Halicyclamine-like Scaffold via Transannular

Building upon their earlier work on DHP dimerization, Wayama and co-workers further advanced the synthetic potential of DHP intermediates by developing a biomimetic transannular cyclization strategy for assembling halicyclamine-type polycyclic scaffolds [36]. While the previous studies demonstrated the feasibility of Brønsted acid-catalyzed dimerization of functionalized DHPs, this subsequent effort explored a more architecturally complex approach to mimic the core connectivity of halicyclamine B. To overcome the limitations of earlier strategies, Wayama’s team designed C_2_-symmetric macrocyclic precursors featuring geminal disubstitution at C6 (to suppress hydride transfer) and electron-withdrawing groups at C3 (to direct regioselective cyclization). A particularly important structural feature was the incorporation of cis-double bonds within the macrocyclic alkyl loops, which induced an oval-shaped conformation. This preorganization aligned the two DHP units in close proximity, thereby facilitating the critical transannular bond formation. Such cis-olefinic geometry is consistent with motifs observed in many natural manzamine alkaloids.

**Scheme 17:**
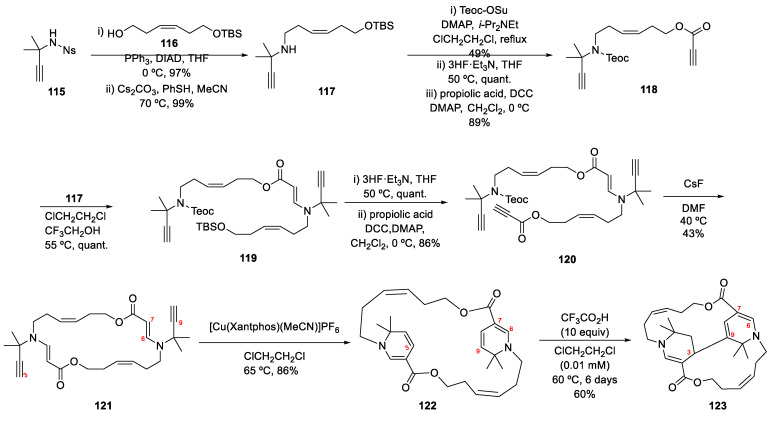
Synthesis of halicyclamine-like scaffold Via transannular cyclization.

As illustrated in Scheme 17, the synthesis began with a dimethylated terminal alkyne **115**, coupled with primary alcohol **116** via a Mitsunobu reaction, followed by nosyl deprotection to provide secondary amine **117**. After Teoc protection and selective TBS deprotection with 3HF·Et_3_N, the resulting alcohol was condensed with propiolic acid to afford half-segment **118**. A heteroconjugate addition between **117** and **118** generated intermediate **119** in quantitative yield, and a second round of deprotection and condensation yielded the other half-segment **120**. Macrocyclization to form **121** was achieved through Teoc deprotection (using CsF in DMF), followed by Cu(Xantphos)(MeCN)PF_6_-catalyzed ring closure, forming bis-1,6-DHP macrocycle **122**. The key transannular cyclization was induced under highly diluted conditions (0.01 μM) in the presence of trifluoroacetic acid at 60 °C for six days, furnishing the halicyclamine-type scaffold **123**. This metal-free, atom-economical, and chemoselective 11-step synthesis represents a powerful realization of a long-speculated biosynthetic hypothesis. It offers an efficient platform for accessing the halicyclamine chemical space, enabling further exploration of TcBPA analogs for both biological and synthetic investigations.

### 4.5. Model Synthetic Studies

**Scheme 18:**
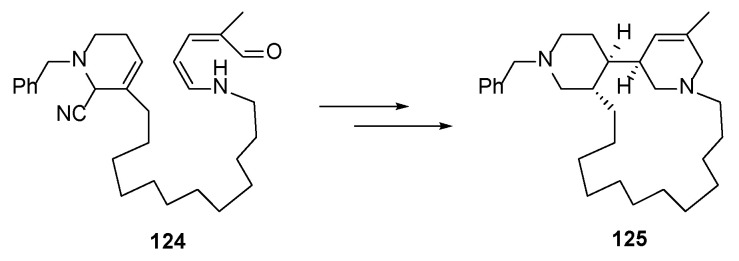
Synthesis of monomacrocyclic halicyclamine A model.

**Scheme 19:**
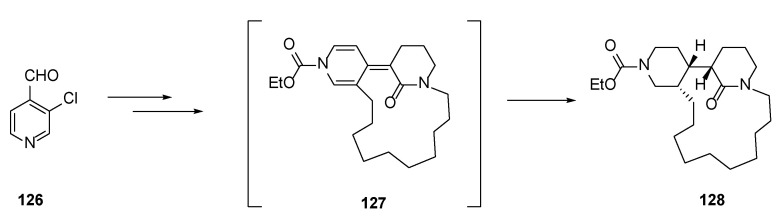
Synthesis of xestoproxamine C analog.

**Scheme 20:**
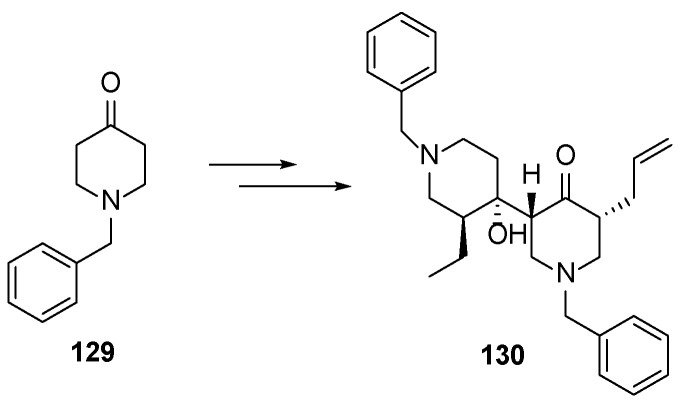
Synthesis of bis(piperidine) derivatives.

**Scheme 21:**
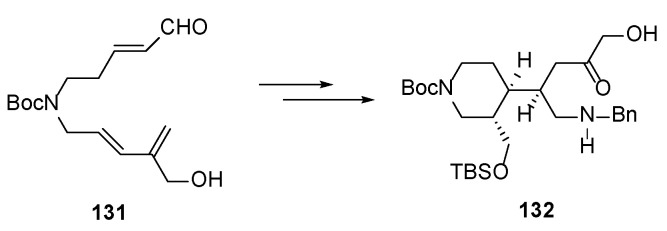
Assembly of the stereogenic triad of the halicyclamine A core.

Efforts to access the complex polycyclic frameworks of TcBPAs have historically relied on model studies designed to probe key structural motifs and test biosynthetic hypotheses. One of the earliest and most influential contributions came from Baldwin, who proposed that manzamine and TcBPA alkaloids could arise via intramolecular Diels–Alder reactions of bis-DHPs or bis-iminium salts (e.g., **34**, **38**, **41**). While this approach led to the successful formation of tetracyclic cores in trace amounts, it was hampered by low yields due to competing disproportionation pathways, driven by intramolecular hydride shifts [32]. Seeking to improve on Baldwin’s concept, Jakubowicz and co-workers [31] developed a series of aminopentadienal-based model reactions (e.g., Scheme 5), exploring their condensation with 5,6-dihydropyridinium salts. Their results demonstrated regioselective formation of TcBPA-like scaffolds with yields superior to earlier methods. Although some products exhibited inverted stereochemistry, the study offered strong support for a modified biosynthetic hypothesis and extended its scope to related frameworks such as sarain A. In a conceptually similar approach, Sinigaglia et al. [39] achieved the formation of a monomacrocyclic halicyclamine A model (**125**) by condensing a 5-aminopenta-2,4-dienal with a masked dihydropyridinium intermediate (Scheme 18). Despite its simplicity, this strategy effectively demonstrated the feasibility of macrocyclization Via aminopentadienal addition, yielding structurally relevant products in moderate yield. Taking a biogenetically inspired angle, Sanchez-Salvatori and Marazano [33] reported a successive condensation sequence involving malondialdehyde derivatives, aldehydes, and primary amines. Their route generated stable bicyclic intermediates resembling the halicyclamine core (Scheme 6). Although anticipated cycloaddition adducts related to manzamine alkaloids were not observed, the ability to access tetraaldehyde-like structures provided rare experimental support for proposed biosynthetic precursors. Meanwhile, efforts to construct bis(piperidine) frameworks through more modular approaches have also been fruitful. Lansakara et al. [7] employed the alkylidene dihydropyridine **127** as an intermediate in a stereoselective synthesis of xestoproxamine C analog (**128**) (Scheme 19), leveraging RCM followed by diastereoselective hydrogenation to achieve controlled assembly of a tricyclic bis(piperidine) motif. Complementing this work, Banwell et al. [40] developed an aldol-based strategy to construct a series of bis(piperidine) derivatives (e.g., **130**), starting from readily available 1-benzyl-4-piperidinone (**129**) (Scheme 20). Their method demonstrated the versatility of α-keto dianion chemistry and yielded unsaturated analogs that preserved biological activity similar to natural haliclonacyclamines, supporting the pharmacological relevance of this substructure. Finally, Molander and colleagues [41] reported a strategic advance in stereocenter construction Via a diastereoselective intramolecular Diels–Alder reaction, aimed at assembling the stereogenic triad (**132**) of halicyclamine A (**16**) (Scheme 21). Although the total synthesis was not completed, the team successfully prepared an advanced intermediate bearing three contiguous stereocenters, establishing a foundation for future synthetic development of TcBPAs.

## 5. Bioactivities and Pharmacological Potential

### 5.1. Anti-Proliferative Activity of TcBPAs on Cancer Cells

**Figure 3:**
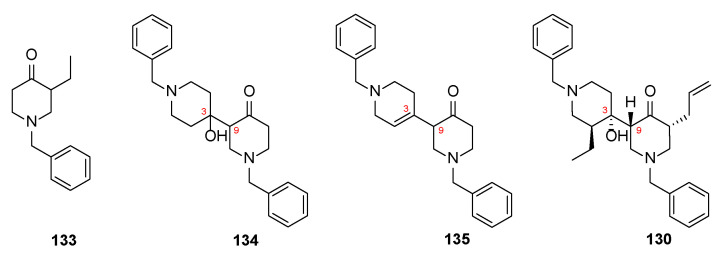
Banwell’s compounds **130**, **133**–**135**.

As illustrated in Table 3 [9,10,12,14,16,18,19,20,21,22,23,24,42], TcBPAs exhibit potent antiproliferative effects across various cancer cell lines, including leukemia, melanoma, breast, colon, fibrosarcoma, and glioblastoma. These compounds typically achieve efficacy at low micromolar concentrations and demonstrate selectivity by sparing healthy cells, as exemplified by neopetrosiamine A (**20**), which shows minimal toxicity toward healthy VERO cells (IC_50_ 96 µM) [14]. It should be noted that all documented IC_50_ values thus far fall within the low micromolar range, highlighting TcBPAs’ potential as candidates for novel leukemia and melanoma therapeutics [43]. This potency may partly result from their macrocyclic structures, enabling interaction with biological targets inaccessible to smaller molecules and traditional treatments [44]. Banwell et al. [40] synthesized and evaluated four open-chain tetra-alkylated 3,9-linked bis-piperidine analogs (**130**, **133**–**135**, Figure 3), which exhibited only moderate inhibition at a single high concentration (20 µM) against thirteen human cancer cell lines. Among these, compound **130**, featuring C-2 and C-7 alkyl substitutions, exhibited superior inhibitory activity against multiple cancer types. These findings suggest that while the 3,9-bis(piperidine) moiety may serve as a pharmacophore, the absence of macrocyclic rings reduces potency, and the length of alkyl substitutions significantly influences biological activity. However, since IC_50_ values were not determined, direct comparison with TcBPAs, whose IC_50_ values fall within the low micromolar range, is limited. Structural differences, including the presence of polar ketone and hydroxyl groups on the core in the intermediates, may further impact their bioactivity and metabolic stability.

Interestingly, the data summarized in Table 3 suggest neither the stereochemistry of the bicyclic core nor the number and location of double bonds significantly impacts the antiproliferative activity. This implies considerable chemical space for optimization and suggests that absolute enantiopurity may not be necessary for effectiveness. Additionally, certain TcBPAs display targeted inhibitory activities against enzymes such as protein kinases and proteasomes, further highlighting their potential as promising anticancer drug candidates.

### 5.2. Antimicrobial Activities

TcBPAs exhibit diverse and promising antimicrobial activities against various pathogens, including bacteria, mycobacteria, fungi, and parasites, making them compelling candidates for anti-infective drug development [34]. Many TcBPAs demonstrate significant antibacterial activity against Gram-positive and Gram-negative bacteria, potentially due to their ability to cross membranes and interact with intracellular targets [30]. Halicyclamine B (**17**) exhibited selective antibacterial activity against *Bacillus subtilis* (50% inhibition at 200 µg/disk) and moderate inhibition against *E. coli* (20% inhibition at 200 µg/disk), and selective antimicrobial activity against *S. aureus* (10 mm inhibition at 100 µg/disk) [11,18]. Acanthocyclamine A (**1**) specifically targeted E. coli and showed selective activity against *S. aureus* with a diameter inhibition of 10 mm at 100 µg/disk [18]. Haliclonacyclamine A (**10**) effectively inhibited *E. coli* (5 mm) and *B. subtilis* (12 mm) at 2.0 µg/mL [40]. Arenosclerins A–C (**2–4**) and haliclonacyclamine E (**14**) displayed broad antibacterial effects but showed no activity against *Candida albicans* [42].

In the context of antimycobacterial activity, halicyclamine A (**16**) demonstrated significant anti-dormant effects against Mycobacterium species under hypoxic and aerobic conditions, with MIC values ranging from 1.0 to 5.0 µg/mL against *M. smegmatis*, *M. bovis* BCG, and *M. tuberculosis* H37Ra [27,34,41]. Haliclonacyclamines A and B (**10**, **11**) exhibited strong antimycobacterial activity against *M. smegmatis* and *M. bovis* BCG (MICs 1.0–2.5 µg/mL) under both aerobic and hypoxic conditions, with haliclonacyclamine B (**11**) appreciably demonstrating bactericidal activity. Conversely, the introduction of a 22-hydroxy group in 22-hydroxyhaliclonacyclamine B (**18**) significantly reduced antimycobacterial efficacy (MICs 12.5–50 µg/mL) [23]. Neopetrosiamine A (**20**) showed potent antituberculosis activity against M. tuberculosis (H37Rv) with MIC values of 7.5 µg/mL, and crude extracts exhibited MICs of 121 and 30 µg/mL. It also displayed potent antiplasmodial activity against Plasmodium falciparum (IC_50_ 2.3 µM) [14].

Regarding antifungal activity, haliclonacyclamine A (**10**) exhibited significant inhibition against Candida albicans (15 mm at 2.0 µg/mL) [40], whereas arenosclerins A–C (**2**-**4**) and halicyclamine B (**17**) showed no activity against this fungus [11,42]. Significant antiplasmodial activities have been observed for haliclonacyclamine A (**10**), which exhibited in vitro potency and in vivo efficacy against Plasmodium vinckei petteri-infected mice and the chloroquine-resistant *P. falciparum* strain FCB1 (IC_50_ values of 0.052 and 0.33 µg/mL, respectively) [11,46], and neopetrosiamine A, with an IC_50_ of 2.3 µM [14]. These structure–activity relationships highlight critical structural features influencing TcBPAs’ antimicrobial effectiveness, underscoring their potential for therapeutic applications. These findings illustrate the range of TcBPAs’ antimicrobial capabilities, implying their potential as therapeutic candidates.

### 5.3. Other Bioactivities of TcBPAs

TcBPAs exhibit significant biological activities beyond antiproliferative and antimicrobial activities, including inhibition of specific protein kinases, proteasome activities, and modulation of amyloid beta-42 production. Acanthocyclamine A uniquely demonstrated inhibition of amyloid β-42 production induced by aftin-5 at a concentration of 26 μM, without cytotoxicity, suggesting the potential of TcBPA scaffold for Alzheimer’s disease intervention [18]. Chloromethylhalicyclamine B was identified as a selective inhibitor of CK1δ/ε kinase, with an IC_50_ value of 6 μM [16]. Structural analyses indicated the essential role of the tetrahydropyridine moiety for CK1δ/ε kinase inhibitory activity, as observed through comparisons with chloromethyltetrahydrohalicyclamine B. Docking studies supported the interaction of chloromethylhalicyclamine B with the ATP-binding site of CK1δ/ε, despite its non-planar structure [16,18].

In proteasome inhibition assays, halicyclamine B demonstrated substantial inhibitory potency against both constitutive and immunoproteasome activities (IC_50_ values: 0.42, 6.3, and 0.48 µM for chymotrypsin-like, trypsin-like, and caspase-like activities, respectively). This potency was notably higher compared to tetrahydrohalicyclamine B. Tetradehydrohalicyclamine B, featuring a pyridinium ring, exhibited markedly lower proteasome inhibition, with minimal or no activity even at higher concentrations, suggesting the pyridinium ring reduces proteasome inhibitory capability [22]. These findings highlight TcBPAs’ potential as valuable leads in developing selective inhibitors for therapeutic interventions in neurodegenerative diseases, cancer, and autoimmune disorders.

## 6. Future Directions and Challenges

TcBPAs constitute a structurally distinctive and biologically intriguing class of marine natural products, holding considerable promise as potential anticancer agents. Nevertheless, despite substantial advancements in their structural elucidation and synthetic accessibility, several critical challenges continue to impede their translational development. Chief among these is the limited natural availability of TcBPAs. Currently, most TcBPAs are sourced through extraction from marine sponges—a method that is environmentally unsustainable and inherently inefficient. Extraction yields are notably low; for instance, haliclonacyclamine A has been isolated at yields as minimal as 0.0005% from sponge biomass [9,10,23,35], while haliclonacyclamine D has been obtained at even lower yields of around 0.0004% from frozen samples [12]. These extremely low yields significantly restrict the availability of pure compounds required for comprehensive biological studies. The scarcity of these alkaloids has consequently limited systematic biological evaluation, particularly regarding in vivo activity. Although several TcBPAs exhibit promising in vitro bioactivity, detailed in vivo studies remain sparse [7]. Consequently, the understanding of their structure–activity relationships (SAR)—including bis-piperidine stereochemistry, macrocyclic topology, and the positioning of olefinic bonds—is incomplete. Expanding biological testing to encompass mechanistic, cellular, and animal studies is essential to fully evaluate therapeutic potential and to guide the rational optimization of analogs.

To effectively overcome supply limitations and accelerate biological assessment, the development of efficient and environmentally sustainable synthetic strategies is crucial. Significant synthetic milestones have already been achieved, including the total synthesis of haliclonacyclamine C and tetrahydrohaliclonacyclamine A by Smith and Sulikowski [38], and a metal-free transannular cyclization approach recently introduced by Wayama. However, many current synthetic methodologies suffer from low overall yields. Advancing synthetic methods toward higher efficiency and sustainability will greatly enhance the accessibility of TcBPAs. Moreover, emerging synthetic technologies such as photoredox catalysis, ring-closing alkyne metathesis, and biomimetic dimerization or transannular cyclization offer potent tools to expand TcBPA chemical diversity. These innovative strategies not only facilitate access to natural products but also create versatile platforms for the rational design and synthesis of analogs, deepening the exploration of SAR in a modular and systematic fashion.

In conclusion, overcoming the dual challenges of limited supply and incomplete biological characterization demands a collaborative interdisciplinary effort. Future research directions should prioritize scaling sustainable synthetic routes, enhancing SAR-driven medicinal chemistry, performing rigorous biological evaluations, and leveraging synthetic advancements for optimized analog design. Such focused efforts will be instrumental in fully realizing the therapeutic potential of this promising, yet underexplored, class of marine alkaloids.

## Figures and Tables

**Table 1:** Natural Sources and Collection Locations of 29 TcBPAs.

Comp. Number	Name	Source Organism	Collection Location	Ref
**1**	Acanthocyclamine A	*Acanthostrongylophora ingens*	South Sulawesi, Indonesia	[17,18]
**2**	Arenosclerin A	*Arenosclera brasiliensis*	Endemic to the Southeastern Brazilian coast	[10]
**3**	Arenosclerin B	*Arenosclera brasiliensis*	Endemic to the Southeastern Brazilian coast	[10]
**4**	Arenosclerin C	*Arenosclera brasiliensis*	Endemic to the Southeastern Brazilian coast	[10]
**5**	Arenosclerin D	*Pachychalina alcaloidifera*	Brazilian	[9]
**6**	Arenosclerin E	*Pachychalina alcaloidifera*	Brazilian	[9]
**7**	Chloromethyltetradehydrohalicyclamine B	*Acanthostrongylophora ingens*	South Sulawesi, Indonesia	[18]
**8**	Chloromethylhalicyclamine B	*Acanthostrongylophora ingens*	South Sulawesi, Indonesia	[16,18]
**9**	Halichondramine	*Halichondria* sp., Unknown species	Red Sea, Dahlak archipelago, Eritrea	[19]
**10**	(−)-Haliclonacyclamine A	*Haliclona* sp. Unknown species	Heron Island, Great Barrier Reef	[20]
**11**	(+)-Haliclonacyclamine B	*Haliclona* sp. Unknown species	Heron Island, Great Barrier Reef	[20]
**12**	Haliclonacyclamine C	*Haliclona* sp. Unknown species	Heron Island, Great Barrier Reef	[12]
**13**	Haliclonacyclamine D	*Haliclona* sp. Unknown species	Heron Island, Great Barrier Reef	[12]
**14**	Haliclonacyclamine E	*Arenosclera brasiliensis*	Endemic to the Southeastern Brazilian coast	[10]
**15**	Haliclonacyclamine F	*Pachychalina alcaloidifera*	IIha do Pai, Niteroi, Rio de Janerio	[9]
**16**	Halicyclamine A	*Haliclona* sp. Unknown species *Amphimedon* sp. Unknown species	Biak, Indonesia Southern Japan	[8,21]
**17**	(−)-Halicyclamine B	*Xestospongia* sp. *Acanthostrongylophora ingens*	Sangihe Islands, Indonesia South Sulawesi, Indonesia	[11,18,22]
**18**	22-hydroxyhaliclonacyclamine B	*Halichondria* sp. Unknown species	Flores Island, Indonesia	[23]
**19**	22-hydroxyhalicyclamine A	*Amphimedon* sp. Unknown species	Southern Japan	[21]
**20**	Neopetrosiamine A	*Neopetrosia proxima*	Mona Island, Puerto Rico	[14]
**21**	Tetradehydrohalicyclamine A	*Amphimedon niphatidae*	Lojima Island, Southern Japan	[21]
**22**	2-*epi-*Tetradehydrohaliclonacyclamine A	*Halichondria* sp. Unknow species	Tulamben Bay, Bali, Indonesia	[24]
**23**	Tetradehydrohaliclonacyclamine A mono-*N*-oxide	*Halichondria* sp. Unknown species	Tulamben Bay, Bali, Indonesia	[24]
**24**	Tetrahydrohaliclonacyclamine A	*Halichondria* sp. Unknown species	Tulamben Bay, Bali, Indonesia	[24]
**25**	Tetradehydrohalicyclamine B	*Acanthostrongylophora ingens*	South Sulawesi, Indonesia	[18,22]
**26**	*epi*-Tetradehydrohalicyclamine B	*Acanthostrongylophora ingens*	South Sulawesi, Indonesia	[18]
**27**	Xestoproxamine A	*Neopetrosia proxima*	Bahamas	[13]
**28**	Xestoproxamine B	*Neopetrosia proxima*	Bahamas	[13]
**29**	Xestoproxamine C	*Neopetrosia proxima*	Bahamas	[13]

**Table 2:** Reported [a]_D_ Values and Configurations for 30 TcBPAs.

Bis-piperidine Alkaloids	[a]_D_ Value	C, g/100 mL	Solvent	Absolute Configurations	Reference
Acanthocylamine A (**1**)	−26.5	0.073	CHCl_3_	2*R*,3*R*,7*R*,9*R* (X-ray anomalous dispersion)	[17,18]
Arenosclerin A (**2**)	−3.0	0.015	MeOH	2*R*,3*S*,7*S*,9*S* (ECCD of bis-P-Br-phenacyl derivative and linked its configuration to that of (-)-perhaliclonacyclamine)	[10,13]
Arenosclerin B (**3**)	+8.6	0.008	MeOH	2S*,3S*,7R*,9R* (Relative configuration)	[10]
Arenosclerin C (**4**)	−17	0.02	MeOH	2S*,3R*,7S*,9S* (Relative configuration)	[10]
Arenosclerin D (**5**)	+6.9	0.014	MeOH	2R*,3S*,9R* (Tentative Relative configuration) The configuration at C7 was not defined.	[9]
Arenosclerin E (**6**)	+14.5	0.015	MeOH	2R*,3S*,7S*,9S* (Relative configuration)	[9]
Chloromethyltetradehydro-halicyclamine B (**7**)	N/A	N/A	N/A	N/A	[18]
Chloromethylhalicyclamine B (**8**)	−94	0.05	MeOH	2*S*,3*R*,7*R* (ECD)	[16]
Halichondramine (**9**)	+3.3	0.54	MeOH	2R*,3S*,7R*,9R* Or 2R*,3S*,7S*,9S* (Relative configuration)	[19]
Haliclonacyclamine A (**10**)	−3.4	1.21	CH_2_Cl_2_	2*R*,3*R*,7*R*,9*R* (X-Ray anomalous dispersion)	[20,24]
Haliclonacyclamine B (**11**)	+3.4	0.55	CH_2_Cl_2_	2*R*,3*R*,7*R*,9*R* (X-ray anomalous dispersion)	[20,24]
Haliclonacyclamine C (**12**)	+4.8	0.30	CH_2_Cl_2_	2*R*,3*R*,7*R*,9*R* (inferred from optical rotation comparison with hydrogenated analogs.)	[12]
Haliclonacyclamine D (**13**)	+16.1	0.30	CH_2_Cl_2_	2*R*,3*R*,7*R*,9*R* *(*NOESY data suggested the configurations; insufficient material for hydrogenation.	[12]
Haliclonacyclamine E (**14**)	+14	0.02	MeOH	2*R*,3*S*,7*S*,9*S* (ECCD of bis-P-Br-phenacyl derivative and linked its configuration to that of (-)-perhaliclonacyclamine)	[10,13]
Haliclonacyclamine F (**15**)	+5.4	0.0041	MeOH	2R*,3S*,7R*,9R* (Tentative Relative configuration) The configuration for ring B was proposed.	[9]
Halicyclamine A (**16**)	−7.3	0.72	CH_2_Cl_2_	2R*,3S*,9R* (Relative configuration)	[8]
	−24.0	0.1	MeOH	2R*,3S*,9R* (Relative configuration)	[21]
Halicyclamine B (**17**)	−143.5	0.65	N/A	2*S*,3*R*,7R Relative configuration (X-Ray); Absolute configuration (ECD)	[11,22]
22-Hydroxyhaliclonacyclamie B (**18**)	+11.8	0.1	MeOH	2*R*,3*R*,7*R*,9*R* (relative configurations were assigned based on ROESY data.) The configuration at C-22 (22-OH) remains undetermined.	[23]
22-Hydroxyhalicyclamine A (**19**)	+21.0	0.1	MeOH	2R*,3S*,9R* (Relative configuration only) No ROESY. Only comparison with ^1^H and ^13^C NMR data.	[21]
Neopetrosiamine A (**20**)	−10.0	1.0	CHCl_3_	2R*,3R*,7R*,9R* (Relative configuration)	[14]
Tetradehydrohaliclonacyclamine A (**21**)	+19.4	0.515	CHCl_3_	2*S*,3*S*,7*S*,9*S* (X-ray anomalous dispersion)	[24]
2-*epi*-Tetradehydrohaliclonacyclamine A (**22**)	+6.6	0.083	CHCl_3_	2*R*,3*S*,7*S*,9*S*	[24]
Tetradehydrohaliclonacyclamine A mono-*N*-oxide (**23**)	+9.2	0.035	CHCl_3_	2*S*,3*S*,7*S*,9*S*	[24]
Tetradehydrohalicyclamine A (**24**)	−14.7	0.1	MeOH	2R*,3S* (Relative configuration)	[21]
Tetradehydrohalicyclamine B (**25**)	N/A	N/A	N/A	2*S*, 3*R* Bioinspired relationship with halicyclamine B ECD	[18,22]
*3-epi*-Tetradehydrohalicyclamine B (**26**) ^a^	N/A	N/A	N/A	2*S*, 3*S* Based on comparison with **26**.	[18]
Xestoproxamine A (**27**)	+4.4	2.0	MeOH	2*R*,3*S*,7*S*,9*S* ECCD	[13]
Xestoproxamine B (**28**)	+2.7	2.4	MeOH	2*R*,3*S*,7*S*,9*S* ECCD	[13]
Xestoproxamine C (**29**)	−18.5	0.67	CHCl_3_	2*S*,3*S*,7*S*,9*S*,*23S* ECCD	[13]
Perhydrohaliclonacyclamine (**30**) ^b^	−20.9	0.205	CHCl_3_	2*S*,3*S*,7*S*,9*S* (X-ray of its parent compound)	[13]

^a^ Epimer **26** was not isolated in pure form; it was obtained only as mixtures enriched in either **25** or **26** [15]. ^b^ This alkaloid has not been isolated from nature yet. N/A: no data was reported.

**Table 3:** Antiproliferative potency of TcBPAs.

Compound	IC_50_ Value in µM (Cell Line)	Cell model	Ref.
Acanthocyclamine A (**1**)	ND	ND	[17,18]
Arenosclerin A (**2**)	8.9 (HL60)	leukemia	[10,42]
	3.6 (B16)	melanoma	
	2.1 4.8 (L929)	fibrosarcoma	
	7.9 (U138)	glioblastoma	
	4.7 (HL60)	leukemia	
Arenosclerin B (**3**)	8.4 (HL60)	leukemia	[10,42]
	3.6 (B16)	melanoma	
	4.6 (L929)	fibrosarcoma	
	7.5 (U138)	glioblastoma	
Arenosclerin C (**4**)	7.5 (HL60)	leukemia	[10,42]
	3.5 (B16)	melanoma	
	4.5 (L929)	fibrosarcoma	
	7.4 (U138)	glioblastoma	
Arenosclerin D (**5**)	12.2 (SF295)	glioblastoma	[9]
	2.4 (MDA-MB435)	reported as breast cancer ^a^	
	12.8 (HCT8)	colon cancer	
	4.3 (HL60)	leukemia	
Arenosclerin E (**6**)	18.0 (SF295)	glioblastoma	
	6.4 (MDA-MB435)	reported as breast cancer ^a^	
	>51.8 (HCT8)	colon cancer	
	14.3 (HL60)	leukemia	
Chloromethyltetradehydro- halicyclamine B (**7**)	ND	ND	[18]
Chloromethylhalicyclamine B (**8**)	ND	ND	[16,18]
Halichondramine (**9**)	ND	ND	[19]
Haliclonacyclamine A (**10**)	1.71 (P388)	lymphocytic leukemia	[12,20,24]
Haliclonacyclamine B (**11**)	1.28 (P388)	lymphocytic leukemia	
Haliclonacyclamine C (**12**)	1.49 (P388)	lymphocytic leukemia	[12,24]
Haliclonacyclamine D (**13**)	N/A	N/A	
Haliclonacyclamine E (**14**)	9.0 (HL60)	leukemia	[10,42]
	3.9 (B16)	melanoma	
	8.3 (L929)	fibrosarcoma	
	13.0 (U138)	glioblastoma	
Haliclonacyclamine F (**15**)	4.5 (SF295)	glioblastoma	[9]
	1.0 (MDA-435)	reported as breast cancer ^a^	
	2.2 (HL-60)	Leukemia	
	8.6 (HCT8)	Colon	
Halicyclamine A (**16**)	0.97 (P388)	lymphocytic leukemia	[21]
(-)-Halicyclamine B (**17**)	12 (HeLa)	Cervical cancer	[22]
22-Hydroxyhaliclonacyclamine B (**18**)	ND	ND	[23]
22-Hydroxyhalicyclamine A (**19**)	0.94 (P388)	lymphocytic leukemia	[21]
Neopetrosiamine A (**20**)	1.5 (MALME-3M)	melanoma	[14]
	2.0 (CCRF-CEM)	leukemia	
	3.5 (MCF)	breast cancer	
	96 (VERO)	African green monkey kidney (normal cell model)	
Tetradehydrohaliclonacyclamine A (**21**)	3.88 (P388)	Lymphocytic leukemia	[24]
2-*Epi*-tetradehydrohalicyclamien A (**22**)	ND	ND	[21]
Tetradehydrohalicyclamine A mono-*N*-oxide (**23**)	ND	ND	[21]
Tetradehydrohalicyclamine A (**24**)	4.8 (P388)	Lymphocytic leukemia	[21]
Tetradehydrohalicyclamine B (**25**)	>50 (HeLa)	Cervical cancer	[22]
Epi-Tetradehydrohalicyclamine B (**26**)	ND	ND	[18]
Xestoproxamine A (**27**)	21.2 (HCT-116)	Colorectal cancer	[16]
Xestoproxamine B (**28**)	6.3 (HCT-116)	Colorectal cancer	[16]
Xestoproxamine C (**29**)	5.4 (HCT-116)	Colorectal cancer	[16]

^a^ MDA-MB-435 cell line was originally described as a human breast cancer cell line. However, its gene expression analysis suggests its melanoma origin instead of breast. MDA-MB-435 cell line was reported to be cross-contaminated with M14 melanoma line [45]. ND: Not determined.

## Abbreviations

The following abbreviations are used in this manuscript:

CD	Circular dichroism
1,6-DHP	1,6-dihydropyridine
DIBAL-H	Diisobutylaluminum hydride
DMF	Dimethylformamide
DMPU	N,N’-dimethylpropyleneurea
ECD	Electronic circular dichroism
ECDD	Exciton-coupled circular dichroism
LDA	Lithium diisopropylamide
LiHMDS	Lithium hexamethyldisilazide
NaBH4	Sodium borohydride
NaH	Sodium hydride
NaHMDS	Sodium hexamethyldisilazide
RCAM	Ring-closing alkyne metathesis
RCM	Ring-closing metathesis
SAR	Structure–activity relationship
TBAF	Tetra-n-butylammonium fluoride
TcBPAs	Tetracyclic bis-piperidine alkaloids
TFA	Trifluoacetic acid
TFAA	Trifluoroacetic anhydride
TMDSO	1,1,3,3-tetramethyldisiloxane
